# PTX-loaded, polysorbate 80-functionalized brain-targeting pullulan nanoparticles for drug delivery

**DOI:** 10.3389/fphar.2025.1666997

**Published:** 2026-01-13

**Authors:** Huabing Yuan, Lu Han, Hao Deng, Yue Tan, Yi Yang, Yi Liu, Jiaxin Ying, Mengqi Fang, Hui Wei, Zhihe Tao

**Affiliations:** 1 The First People’s Hospital of Tianmen in Hubei Province, Tianmen, China; 2 Medical College Wuhan University of Science and Technology, Wuhan, China; 3 Hunan Normal University School of Medicine, Changsha, China

**Keywords:** brain targeting, drug delivery, polysorbate 80, pullulan, self-assembly

## Abstract

Brain-targeted drug delivery remains a major challenge in pharmaceutical research. In this study, four types of polysorbate 80 and cholesterol-modified pullulan polymers (CHPP) were synthesized and self-assembled into nanoparticles (CHPP NPs) for brain drug delivery. The properties of these NPs, including brain targeting efficiency, were systematically evaluated to investigate the influence of polymer modification on their performance. The hydrophobicity of the polymers increased with the degree of cholesterol substitution, which in turn led to a reduction in nanoparticle size. Furthermore, higher hydrophobicity contributed to an increased drug loading capacity for paclitaxel (PTX) and a more sustained drug release profile. *In vivo* fluorescence imaging revealed that formulations with higher levels of polysorbate 80 and cholesterol modification exhibited significantly enhanced brain targeting efficiency compared to other variants. The PTX-loaded CHPP NPs (PTX-CHPP NPs) demonstrated potent cytotoxicity and inhibitory effects against SJ-GBM2 glioma cells *in vitro*, underscoring their potential as a promising platform for brain-targeted drug delivery. These findings provide valuable insights for the rational design of brain-targeting nanocarriers.

## 1 Background

Brain tumors have remained a persistent and formidable challenge over the past several decades (Mellinghoff et al., 2023a; Mellinghoff et al., 2023b). One of the most significant challenge is achieving effective delivery of therapeutic agents to the brain. The blood-brain barrier (BBB) takes precise control of entrance and expulsion of the molecules in vascular compartment to the brain (Wu et al., 2023/05). It could excludes more than 98% of small molecule drugs and almost all macromolecular therapeutics from reaching the brain parenchyma (Pandit et al., 2020/01; Banks, 2016/04).

Although the nanotechnology has obtained a huge development for cancer diagnosis and therapy, there is still no significant progress in the treatment of the brain tumors (Karimi et al., 2023; Cui et al., 2023). The key challenge remains how to deliver drugs directly to the brain with minimum toxic effects. To overcome the challenge, several novel technologies have been developed and exhibited promising potential for clinical use, such as the utilization of nanoparticles drug delivery system (Tiwari et al., 2023; Miao et al., 2023; Liao et al., 2023). The nanoparticles drug delivery system can provide more precise and focused treatment, and improve the potential therapeutic via enhancing the effectiveness and reducing the toxicity (Dilliard and Siegwart, 2023). Currently, nanoparticles (NPs) with surface modification, such as brain-targeting ligand and cell membrane coating, have been widely researched for the treatment of brain tumors (Tylawsky et al., 2023; Ma et al., 2023). Kaicheng Tang et al. proposed an “allosteric targeting” strategy: which would achieve precise brain targeting property by the construction of a “plug-and-play” lipid-based drug delivery system (Tang et al., 2025/04). NPs with the modification of polysorbate 80 on the surface can adsorb apolipoprotein E (ApoE) in the process of plasma transport (Joseph et al., 2021; Tao et al., 2021). With the assistance of ApoE, the NPs could be recognized and taken up by the blood brain barrier endothelial cells via the LDL receptor mediated endocytosis and hence, achieve the brain targeting property (Yusuf et al., 2021; Zensi et al., 2009). The density of polysorbate 80 modified on the surface of the NPs may play a paramount role on their brain drug delivery property (Blasi et al., 2007).

Polymer nanoparticles are a commonly used nanoparticle delivery system that are self-assembled from amphiphilic polymers (Muraoka et al., 2022; Agrawal et al., 2022). The hydrophobic group in the polymer assembles into the hydrophobic core of the nanoparticle, while the hydrophilic group forms the hydrophilic shell (Zhao et al., 2023). Hence, the amphipathy of the polymers could influence the properties of the NPs, such as the size, the zeta potential, the drug release and the biodistribution (Tao et al., 2018a; Tao et al., 2018b). To obtain the NPs with proper properties, varieties of functional modifications were made on the polymers (Zong et al., 2023; Ding et al., 2022). For example, the acid-labile hydrazone linkage would provide a pH-sensitive property (Xiao et al., 2024; Wu et al., 2023; Yang et al., 2023). Therefore, it is of great significance to study the effect of the modification of the materials on the performance and function of NPs in order to improve the therapeutic efficacy of the brain drug delivery system.

PTX, a kind of diterpenoid alkaloid compound, is used as a broad-spectrum anticancer drug in the clinical treatment of head and neck cancer (Zhou et al., 2024; Wang F. et al., 2023). However, the highly hydrophobic property make PTX difficult to be absorbed and utilized *in vivo*. As a result, the PTX must be solubilized using surfactants such as Cremophor EL or polysorbate 80 before intravenous administration. To address the problem, PTX loaded nanoparticles drug delivery system (NDDS) with enhanced delivery efficiency were prepared, and the targeting property also prevented drug diffusion to the nontarget tissues in the blood circulation and reduced the systemic side effects (Wang K. et al., 2023; Li et al., 2024; Jiang et al., 2022).

**FIGURE 1:**
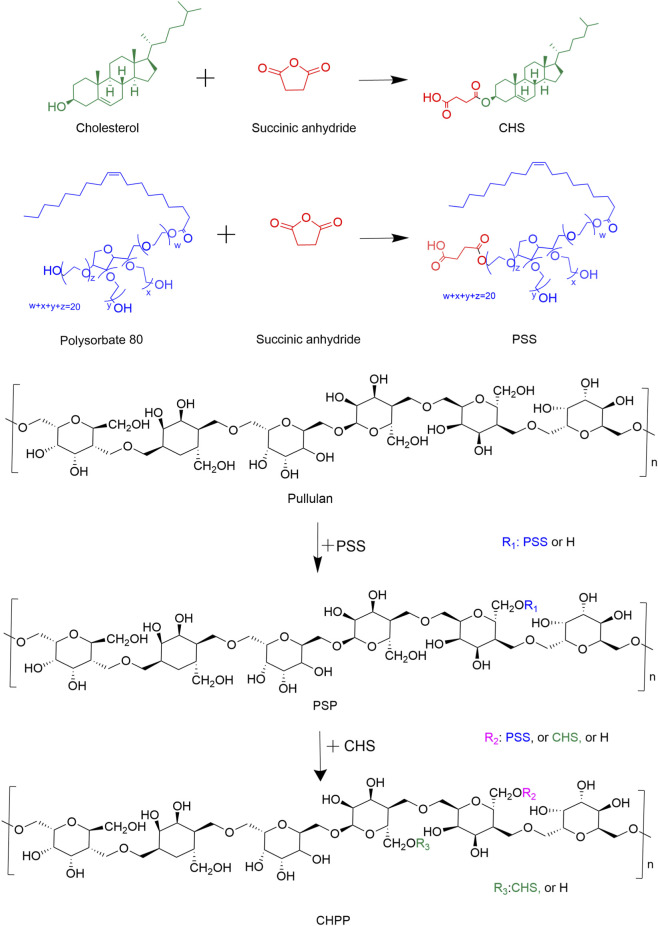
Synthesis route of the CHPP conjugate.

Pullulan polysaccharide is a kind of biological polysaccharide composed of a large number of hydroxyl groups and is commonly used as a drug carrier (Duan et al., 2023; Nochi et al., 2010; Gupta and Gupta, 2005). It possesses the advantages of nontoxicity, easy modification, biodegradability and good water solubility. In this paper, the cholesterol was conjugated to pullulan to increase the hydrophobicity of pullulan, and the polysorbate 80 was introduced to provide a brain targeting property (Figure 1). To obtain the optimized property for brain drug delivery, the effect of the modification of pullulan on the performance and function of NPs were explored. This research would provide new directions for the design of polymer-based brain targeting drug delivery system.

## 2 Materials

Polysorbate 80 was obtained from Tianjin HengXing Chemical Reagent. Pullulan (MW 200 kDa), DMEM supplemented with 10% fetal bovine serum (FBS), 4-dimethylaminopyridine (DMAP), and N-hydroxysuccinimide (NHS) were sourced from Shanghai Aladdin Biochemical Technology Co., Ltd. Paclitaxel (PTX) was acquired from Shanghai Aladdin Reagent Co., Ltd. All other analytical-grade chemicals were purchased from Changsha Yancao Commerce Co.

## 3 Methods

### 3.1 Synthesis of polysorbate 80 ester

Succinic anhydride (SA, 1.2 g) was dissolved in 20 mL of DMSO. 4.0510 g of polysorbate 80 was added to the solution, and the reaction was performed at 50 °C for 48 h. The polysorbate 80 ester (PSS) was obtained as grayish-white powders by dialysis and freeze-drying.

### 3.2 Synthesis of polysorbate 80 ester pullulan

Pullulan, PSS, DMAP, and NHS (sugar unit of pullulan: PSS: DMAP: EDC = 1:1:1:1.5 or 1:2:2:3, mmol) were dissolved in DMSO, and reacted at 50 °C for 48 h. Dialysis and freeze-drying were performed to obtain PSP1 and PSP2.

### 3.3 Synthesis of cholesteryl ester

Cholesterol (2.5 g) and succinic anhydride (2.0 g) were put into a flask, and pyridine (20 mL) was slowly added at room temperature. Next, the solution was slowly poured into hydrochloric acid solution (pH 2–3) under stirring and then refrigerated at 4 °C for 2 h. Complete precipitation was followed by filtration. The solids obtained were dissolved in 40 mL of ethyl acetate and 40 mL of ethanol in a water bath and recrystallized for 4 h after refrigeration. Finally, we filtered and dried the solution to obtain CHS.

### 3.4 Synthesis of CHPP

CHS (1.0 mmol, 0.67 mmol and 0.5 mmol) was weighed with corresponding amounts of DMAP and EDC (CHS: DMAP: EDC = 1:1:1.5, mmol), dissolved in DMSO, and activated by stirring at room temperature for 1 h. PSP1 (sugar unit of PSP: CHS = 1:1, 2:3, or 1:2) was added to the solution and reacted at 50 °C for 48 h (Figure 1). After dialysis and lyophilization, CHPP1, CHPP2 and CHPP3 were obtained.

CHS (1.0 mmol), DMAP (0.13 g) and EDC (0.24 g) were weighed, dissolved in an appropriate amount of DMSO, stirred and activated at room temperature for 1 h, and PSP2(sugar unit of PSP: CHS = 1:1) was added to this solution and reacted at 50 °C for 48 h. CHPP4 was obtained after dialysis and lyophilization.

### 3.5 FTIR and H^1^NMR measurements

Small amounts of Pullulan, CHS, PSP, and CHPP samples were measured by a Fourier transform infrared (FTIR) spectrometer (Nicolet NEXUS 470-ESP, United States). All of the samples were dissolved in DMSO-D6 under ultrasound and were measured by H^1^NMR spectra (BRUKER AVANCE-500, Bruker, Billerica, MA, United States).

### 3.6 Preparation of nanoparticles

PTX (10 mg) and CHPP polymers (50 mg) were dissolved in DMSO(5 mL) to used as the organic phase. Then the organic phase was introduced into a dialysis bag (MWCO 8000-14000) and dialyzed against 1,000 mL of water as the aqueous phase. Four types of PTX-loaded nanoparticle solutions were obtained after 24 h.

FITC-loaded PTX nanoparticles were obtained via the addition of FITC (4 mg) in the organic phase during the above process.

### 3.7 DLS measurements and microscopy observation

The nanoparticle solutions were poured into the sample cell and detected by DLS (Zetasizer 3000 HS, Malvern Instruments, Malvern). The nanoparticle solutions were dropped on a copper mesh and placed in a dryer to dry naturally. The nanoparticles were negatively stained by 2% (W/W) phosphotungstic acid and observed by transmission electron microscopy (Tecnai G2 20 S-Twin, FEI Hong Kong Inc., Hong Kong, China). The nanoparticle solutions were added dropwise to a clean silicon wafer, and then placed under a JSM-6700F field emission scanning electron microscope to observe the morphology of PTX-loaded nanoparticles.

### 3.8 Determination of the drug release rate

According to the characteristic absorption peak of PTX at 227 nm, the standard curve of PTX solution was established, which was used for the detection of the PTX. The drug loading of nanoparticles (DL) was calculated using the following formula:

$$\text{DL}\%=\left(\text{the amount of drug in the nanoparticles}\right)/\left(\text{the amount of nanoparticles weight}\right)\times 100\%.$$

The *in vitro* drug release studies of nanoparticles were performed at pH 7.4 and 6.8 via the dialysis technique. The nanoparticles were dispersed in a PBS buffer solution (12 mL) and placed in a pre-swelled dialysis bag (MWCO 3500 Da). Then, the dialysis bag was then immersed in PBS (0.1 M, 150 mL, pH 7.4) and oscillated continuously in a shaker incubator (180 rpm) at 37 °C. All samples were assayed by UV-Vis. The percentage rate of drug release (Q%) was calculated using the following formula:

$$Q\%=\left(C_{n}\times V+V_{n}\sum_{t=0}^{n}C_{i}\right)/\left(W_{NP}\times DL\%\right)$$

where W is the NP weight, C_n_ is the sample concentration at T_n_, V is the total volume of release medium, V_n_ is the sample volume, and C_i_ is the sample concentration at T_i_ (i = 0, 0.5, 1, …… n h, both V_0_ and C_0_ are equal to zero).

### 3.9 Animals and cell cultures

SJ-GBM2 neuroma cells were was obtained from the American Type Culture Collection. The complete growth medium was DMEM supplemented with 10% FBS and 1% penicillin/streptomycin. The cells were cultivated in an incubator (Thermo Scientific) at 37 °C in the presence of 5% CO_2_ for 24 h.

C57BL/6 mice (5–6 weeks, 16–20 g) were purchased from Shanghai Laboratory Animal Center, Chinese Academy of Sciences.

### 3.10 *In vivo* fluorescence imaging

Sixteen C57BL/6 mice were randomly divided into 4 groups, namely, FITC-labeled CHPP1 group, FITC-labeled CHPP2 group, FITC-labeled CHPP3 group and FITC-labeled CHPP4 group. FITC-labeled CHPP NPs with equivalent fluorescence intensities were intravenously administered into the C57BL/6 mice via tail veins. At 6 h post-injection, the mice were anesthetized and imaged *in vivo* with the Maestro imaging system (IVIS Lumina LT, PerkinElmer).

### 3.11 Cell viability

SJ-GBM2 neuroma cells were cultured in 96-well plates at a density of 5 × 10^3^ cells/well. After 24 h, four kinds of CHPP nanoparticles loaded with PTX and free PTX were added to make the concentration of PTX 0.25 μg/mL, 0.5 μg/mL, 1 μg/mL and 2 μg/mL, respectively. After 12 h of culture at 37 °C, the culture medium was taken and 20 μL of MTT solution (5 μg/mL) dissolved in PBS was added. After incubation for 4 h, 150 μL DMSO was added and shaken for 10 min. The absorbance value (A) at 490 nm was determined by microplate reader.

### 3.12 Statistical analysis

The statistical significance of treatment outcomes was assessed using one-way/two-way analysis of variance for the differences within treatments followed by Tukey’s *post hoc* test (Prism 7 for Windows, GraphPad Software Inc., United States); P < 0.05 was considered statistically significant in all analyses (95% confidence level).

## 4 Results and discussion

**FIGURE 2:**
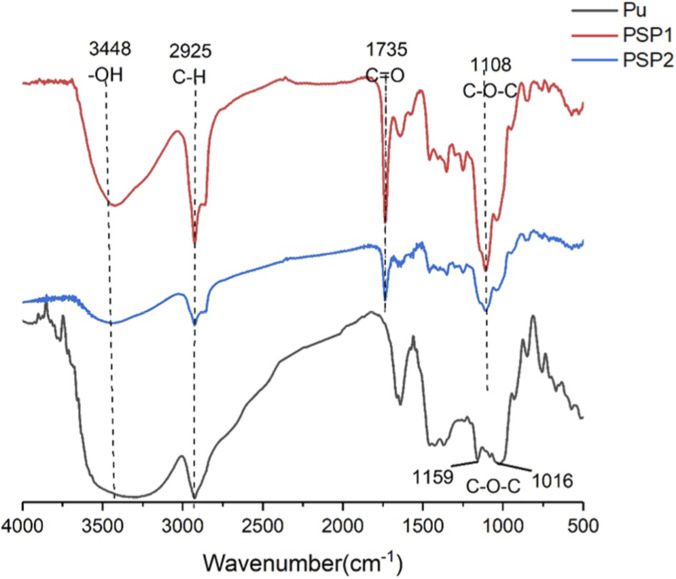
The FT-IR spectra of Pullulan (Pu), PSP1 and PSP2.

In order to enhance the brain targeting effect of the NDDS, pullulan nanoparticles with the modification of polysorbate 80 were prepared. Firstly, polysorbate 80 conjugated pullulan (PSP) was synthesized via esterification reactions (Figure 1). Compared with the pullulan molecules, the peaks around 3,448 cm^-1^ were significantly decreased in the FT-IR spectra of the PSP, illustrating that part of the oxhydryl groups in the pullulan molecules had been modified (Figure 2). And two new adsorption peaks appeared at 1735 cm^−1^ and 1,108 cm^−1^, which were assigned to the C=O stretching vibration and C-O-C stretching vibration of the newly formed ester bond, respectively. These results confirmed the successful preparation of the PSP conjugation.

Under different feeding ratios of polysorbate 80 to pullulan, PSP1 and PSP2 were obtained. Compared with PSP1, the -OH stretching vibration peak at 3,448 cm^−1^ in the IR spectra of the PSP2 were significantly stronger, indicating that more oxhydryl groups were retained and less polysorbate 80 was conjugated.

**FIGURE 3:**
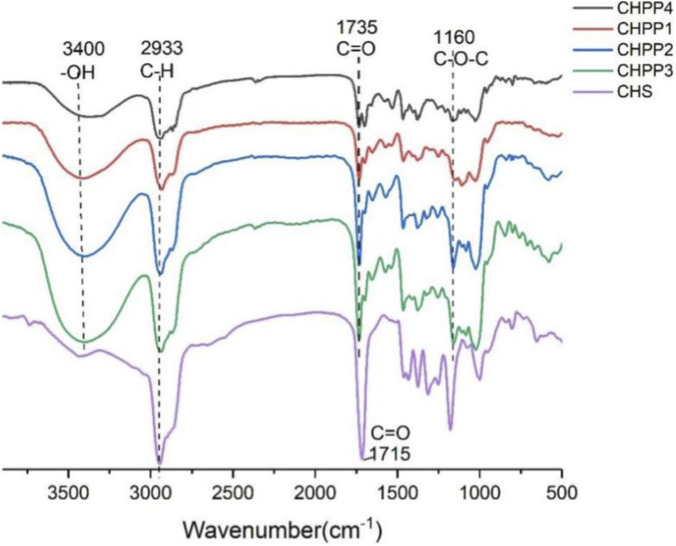
The FT-IR spectra of CHS, CHPP1, CHPP2, CHPP3 and CHPP4.

As a hydrophilic molecule, PSP should be hydrophobically improved before self-assembly. Since the polysorbate 80 targeted to the brain via absorbing ApoE (Joseph et al., 2021; Tao et al., 2021), cholesterol, which possessed good affinity to proteins, was chosen to improved the hydrophobicity of PSP. As shown in Figure 1, succinic anhydride modified cholesterol was conjugated to pullulan via an ester bond. In the reaction process, the degree of substitution of CHS could be controlled by the feeding ratio. With the further modification of pullulan, the peaks around 3,400 cm^−1^ decreased and the peaks at 2,933 cm^−1^ increased significantly in the FT-IR spectra of CHPP (Figure 3), illustrating that the oxhydryl group of the pullulan was conjugated with CHS. Moreover, a shoulder peak emerged alongside with the peak of 1735 cm^−1^, which was ascribed to the newly formed ester bond. And the shoulder peak increased with the feeding ratio of CHS, indicating that more CHS were conjugated to PSP molecules via esterification.

**FIGURE 4:**
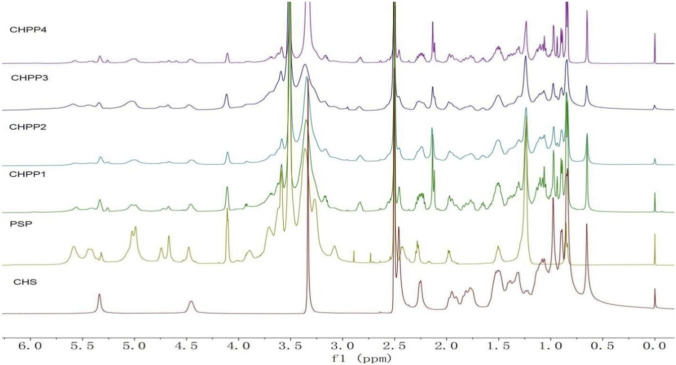
The H^1^NMR spectra of CHS, PSP, CHPP1, CHPP2, CHPP3 and CHPP4.

Then, the H^1^NMR was used to further confirm the structure of the CHPP molecule. As shown in Figure 4, the characteristic peaks of oleic protons the peaks at 1.2 ppm and vinyl protons at 5.0 ppm, corresponding to the polysorbate 80, and peaks of protons at 0.7–1.1, corresponding to the sterane moiety of cholesterin, were evidently observed in the H^1^NMR spectrum of CHPP. These results indicated that CHS and PSP were successfully conjugated to hydroxyl group of pullulan via ester bond.

**FIGURE 5:**
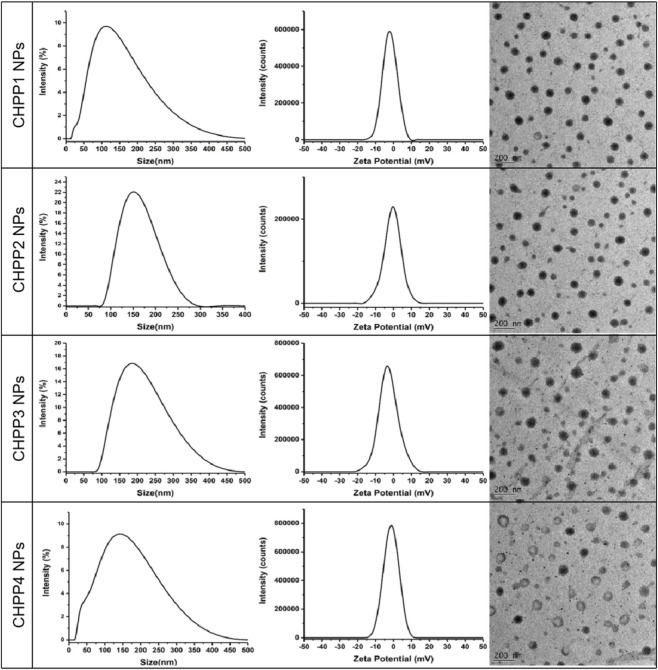
Particle size, potential and TEM graphs of CHPP NPs.

Then, four types of CHPPs were employed to self-assemble into nanoparticles (CHPP1, CHPP2, CHPP3 and CHPP4 NPs) and their properties were systematically characterized. As shown in the TEM images (Figure 5), the CHPP NPs were in the range of 50–150 nm and regularly spherical in shape. Under the dynamic light scattering, the size of CHPP1, CHPP2, CHPP3 and CHPP4 NPs were 89.87 ± 2.14, 142.7 ± 3.56, 183.3 ± 4.22 and 90.54 ± 2.86 nm (Table 1), indicating that the size of the NPs was determined by the ratio of CHS in the preparation process. When increasing the substitution of CHS, the size of the nanoparticles decreased significantly. Nevertheless, the ratio of PSS had little effect on the size of the nanoparticles. The reason might be that the hydrophobicity of CHPP molecules play a paramount role on the self-assembly process and affecting the particle size, which was directly controlled by the substitution of CHS. The zeta potential of the CHPP NPs were in the range of −2.0–1.0 mV, indicating that it was little affected by the substitution of pullulan.

**TABLE 1:** Characteristics of the CHPP NPs.

Sample	Pullulan:PSS (mmol/mmol)	PSP:CHS (mmol/mmol)	Size (nm)	PDI	Zeta potential (mV)
CHPP1 NPs	2:1	2:1	89.87 ± 2.14	0.252 ± 0.041	–(2.02 ± 0.08)
CHPP2 NPs		3:1	142.7 ± 3.56	0.111 ± 0.022	–(1.58 ± 0.06)
CHPP3 NPs		4:1	183.3 ± 4.22	0.163 ± 0.028	–(1.59 ± 0.06)
CHPP4 NPs	1:1	2:1	90.54 ± 2.86	0.282 ± 0.032	–(1.40 ± 0.05)

**FIGURE 6:**
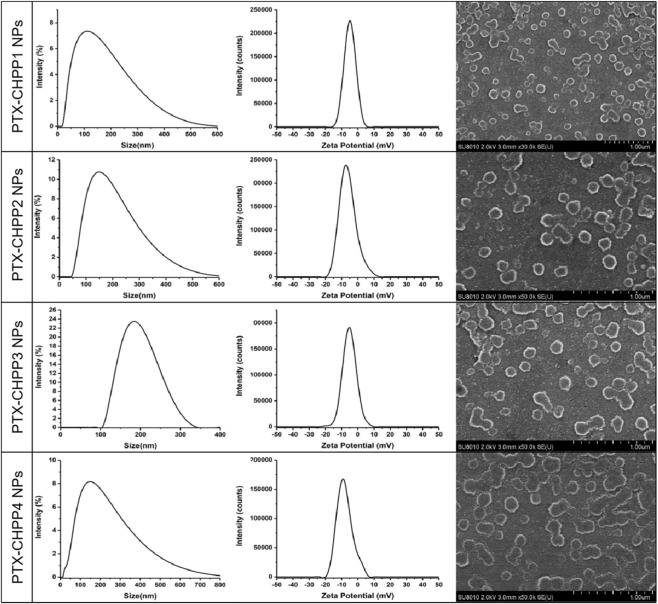
Particle size, potential and scanning electron microscopy images of PTX-loaded CHPP nanoparticles.

To observe the properties of the CHPP NPs for drug delivery, PTX was loaded in the CHPP NPs, and their properties were systematically studied *in vitro* and *in vivo*. As shown in Table 2, the loading of PTX made the NPs slightly larger than before. Moreover, many PTX-CHPP NPs failed to assemble into independent and spherical nanoparticles. Especially in the SEM images of the PTX-CHPP4 NPs, a majority of the nanoparticles were connected the at least one particle (Figure 6). As a strongly hydrophobic molecule, the loading of PTX into the hydrophobic nucleus of the NPs would greatly change the amphiphilic condition of the self-assembly process and affect the formation of the NPs. As a result, the zeta potential of the PTX-CHPP NPs was also influenced and reduced to about −5 mV. The retention of the highly hydrophobic drug candidates has always been a challenge, owing to that they easily precipitated out during the preparation process. Hence, the drug loading of the PTX-CHPP NPs was no more than 7.5%. Since PTX needed a hydrophobic environment, the drug loading increased with the hydrophobicity of CHPP molecules. As a result, the PTX-CHPP1 NPs possessed the highest drug loading of 7.24%.

**TABLE 2:** Characteristics of four types of the drug-loaded nanoparticles.

Sample	Size (nm)	PDI	Zeta potential (mV)	Loading capacity (%)
PTX-CHPP1	102.0 ± 3.05	0.339 ± 0.048	–(4.86 ± 0.72)	(7.24 ± 0.36)%
PTX-CHPP2	152.6 ± 3.60	0.332 ± 0.046	–(5.15 ± 0.85)	(6.58 ± 0.28)%
PTX-CHPP3	208.2 ± 4.36	0.204 ± 0.038	–(5.23 ± 0.88)	(5.84 ± 0.22)%
PTX-CHPP4	111.6 ± 3.16	0.375 ± 0.052	–(5.08 ± 0.78)	(7.05 ± 0.32)%

**FIGURE 7:**
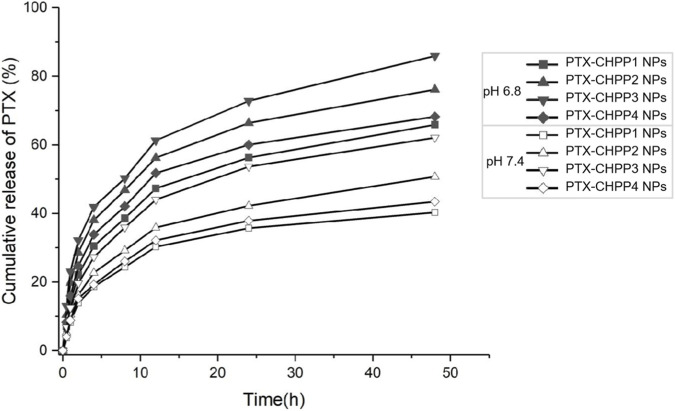
The release profiles of PTX from CHPP nanoparticles at pH 6.8 and 7.4.

The *in vitro* drug release studies of the four types of PTX-CHPP NPs were performed using a dialysis technique under pH 6.8 or 7.4. All samples were assayed by UV spectrophotometry. The release profiles are shown in Figure 7. Since PTX is encapsulated within the core of the nanoparticles, its release profile is directly dependent on the disintegration of the NPs. Due to that the amphipathicity of CHPP resulted from the modification of CHS via esterification, the hydrolytic cleavage of the ester bonds would lead to the disintegration of the NPs and the release of PTX. Hence, the release of PTX from the PTX-CHPP NPs possessed a significantly prolonged and sustained drug release profile. A higher degree of CHS substitution corresponded to a more stable nanoparticle state and a slower drug release profile. As a result, the drug release profiles of PTX-CHPP1 NPs and PTX-CHPP4 NPs were more sustained than those of the other two kinds of NPs. Since ester bonds would be hydrolytic cleaved easier in the acidic condition, the release of PTX was largely quickened under pH 6.8. The result indicated that the PTX-CHPP4 NPs would be most stable in the blood circulation and disintegrate quickly at the tumour site and thus deliver more PTX to the tumour cells.

**FIGURE 8:**
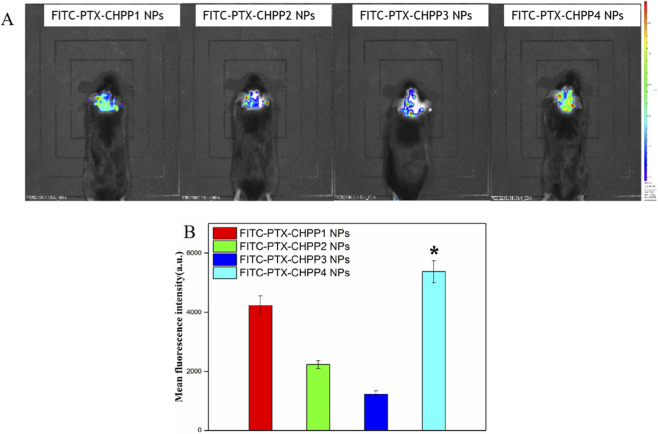
The *in vivo* distribution images **(A)** and their quantification **(B)** of the FITC-PTX-CHPP NPs by small animal imaging system (*P < 0.05, compared to the FITC-PTX-CHPP NPs group).

To explore the brain targeting ability of the PTX-CHPP NPs, the *in vivo* biodistribution of PTX-CHPP NPs was studied. The treatment was performed by injecting the FITC-PTX-CHPP NPs intravenously into the mice. Afterwards, the fluorescent images of the mice were taken at 6 h post-injection to compare the brain targeting effect of the four kinds of the PTX-CHPP NPs. All the mice injected with the NPs possessed an enhanced fluorescence intensity in the brain, suggesting that all kinds of FITC-PTX-CHPP NPs could cross the BBB and enter the brain tissue of C57BL/6 mice (Figure 8). The conjugation with polysorbate 80 provided a brain targeting effect to the NPs. Importantly, the mouse of the FITC-PTX-CHPP4 NPs group possessed the strongest fluorescence intensity in the brain, indicating that the targeting effect was determined by the degree of substitution of polysorbate 80. The higher degree of substitution would enhance the adsorption capacity for apolipoprotein E, induce a better penetration effect to BBB and deliver more drug candidates to the brain. Amnog the other three kinds of NPs with the same degree of substitution of polysorbate 80, their targeting effects were also different. The FITC-PTX-CHPP1 NPs possessed a significantly enhanced targeting effect, which was almost close to that of the FITC-PTX-CHPP4 NPs. The reason might be that their size, Zeta potential, and the drug loading were all different. The smaller size increased the specific surface areas, and more cholesterol in the polymer might increase the affinity to proteins. All the facters might contribute to the absorption of ApoE by the NPs.

**FIGURE 9:**
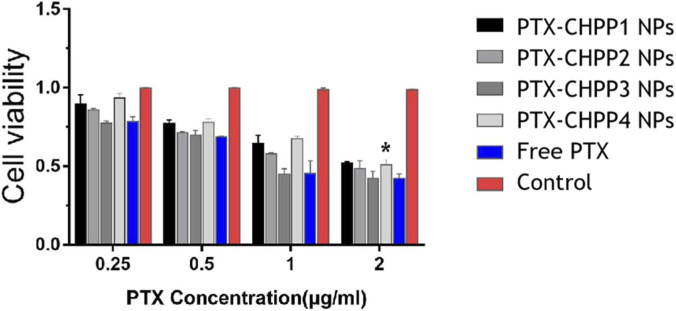
Cell survival rates after 12 h of administration of PTX-CHPP NPs and free PTX (*P < 0.05, compared to free PTX group).

To further investigate the possibility of utilizing the CHPP NPs for drug delivery, the killing ability of the PTX-CHPP NPs to SJ-GBM2 neuroma cells was tested. The concentrations of PTX were 0.25, 0.50, 1.00, and 2 μg/mL. As is shown in Figure 9, the cytotoxicity of the free drug and the PTX-CHPP3 NPs was higher than that of the other NPs, mainly because of the much faster drug release rate than the others. There are two ways for the PTX to take effect: (1) The PTX is released from the NPs outside the cells, and then enters the cells. (2) The NPs are taken up by the cells, and then release the drug inside the cells (Panyam and Labhasetwar, 2003; He et al., 2010; Rui et al., 2022). Although the NPs might effectively enter the cells, it also took time to release the PTX to kill the cells. Hence, the cytotoxicity of the NPs was closely related to their drug release rate in this test. The fast drug release of the PTX-CHPP3 NPs and free PTX resulted in an enhanced killing ability to the SJ-GBM2 neuroma cells. Moreover, the enhanced targeting property of PTX-CHPP1 NPs and PTX-CHPP4 NPs should function via absorbing ApoE *in vivo*. As a result, the PTX-CHPP3 NPs killed the most cells after 12 h co-incubation *in vitro*.

## 5 Conclusion

In summary, we developed brain-targeted nanoparticles based on polysorbate 80 and cholesterol-modified pullulan, which exhibited sustained drug release. Surface decoration with polysorbate 80 was found to be critical for imparting brain-targeting ability, with its efficiency correlating positively with the surface density of polysorbate 80. Additionally, cholesterol modification synergistically enhanced the targeting effect. These findings provide valuable insights for optimizing the design of brain-targeted delivery systems. Future work should focus on evaluating the *in vivo* performance of these CHPP NPs, including systematic studies on their biopharmaceutics, pharmacokinetics, antitumor efficacy, and biocompatibility.

## Data Availability

The original contributions presented in the study are included in the article/supplementary material, further inquiries can be directed to the corresponding authors.
